# SARS-CoV-2 Seroprevalence Compared with Confirmed COVID-19 Cases among Children, Colorado, USA, May–July 2021

**DOI:** 10.3201/eid2905.221541

**Published:** 2023-05

**Authors:** Shannon C. O’Brien, Lyndsey D. Cole, Bernadette A. Albanese, Allison Mahon, Vijaya Knight, Nathan Williams, Rachel Severson, Alexis Burakoff, Nisha B. Alden, Samuel R. Dominguez

**Affiliations:** Colorado Department of Public Health and Environment, Denver, Colorado, USA (S.C. O’Brien, N. Williams, R. Severson, A. Burakoff, N.B. Alden);; Children’s Hospital Colorado, Aurora, Colorado, USA (L.D. Cole, A. Mahon, V. Knight, S.R. Dominguez);; Tri-County Health Department, Greenwood Village, Colorado, USA (B.A. Albanese)

**Keywords:** COVID-19, 2019 novel coronavirus disease, coronavirus disease, severe acute respiratory syndrome coronavirus 2, SARS-CoV-2, SARS-CoV-2 infection, viruses, respiratory infections, zoonoses, 2019 novel coronavirus infection, 2019-nCoV, pandemic, serologic testing, antibody testing, diagnostic testing, Colorado, United States

## Abstract

To compare SARS-CoV-2 antibody seroprevalence among children with seropositive confirmed COVID-19 case counts (case ascertainment by molecular amplification) in Colorado, USA, we conducted a cross-sectional serosurvey during May–July 2021. For a convenience sample of 829 Colorado children, SARS-CoV-2 seroprevalence was 36.7%, compared with prevalence of 6.5% according to individually matched COVID-19 test results reported to public health. Compared with non-Hispanic White children, seroprevalence was higher among Hispanic, non-Hispanic Black, and non-Hispanic other race children, and case ascertainment was significantly lower among Hispanic and non-Hispanic Black children. This serosurvey accurately estimated SARS-CoV-2 prevalence among children compared with confirmed COVID-19 case counts and revealed substantial racial/ethnic disparities in infections and case ascertainment. Continued efforts to address racial and ethnic differences in disease burden and to overcome potential barriers to case ascertainment, including access to testing, may help mitigate these ongoing disparities.

Since identification of SARS-CoV-2 and the ensuing pandemic, epidemiologic studies have shown that COVID-19 outcomes are less severe among children than adults (*1*–*8*). Conclusions drawn from published studies on COVID-19 prevalence in the pediatric population have varied (*5*,*9*–*16*), possibly because of differences in mitigation measures, community transmission rates, and case ascertainment practices. Noted trends in the prevalence of SARS-CoV-2 infection among children can be affected by population-level decisions, such as viral test prioritization and distribution as well as individual-level test-seeking behavior, because the disease in many children is asymptomatic or only mildly symptomatic. In addition, societal effects, such as loss of work for parents and caregivers and missed school days if a child tests positive, may also affect test-seeking behavior. Those factors can lower case ascertainment when relying on reported viral testing as the primary source of surveillance data, resulting in underestimation of disease burden and transmission in the pediatric population, and may lead to an overestimation of disease severity (*17*).

The issue of underestimation by disease surveillance systems that rely heavily on diagnostic testing and reporting is not specific to SARS-CoV-2. Strategies to improve the accuracy of disease prevalence estimates exist, including use of mathematical modeling. However, modeling based on more consistently reported outcome-based metrics, such as hospitalizations, can be limited in the ability to describe disease prevalence in some subpopulations, especially when measured outcomes are rare, as with SARS-CoV-2 hospitalizations among children. Thus, other modalities are needed to refine information about SARS-CoV-2 prevalence among children.

True prevalence of pediatric SARS-CoV-2 infection may be more accurately measured by using laboratory assays to detect SARS-CoV-2 IgG, compared with surveillance systems that rely on viral testing (*17*,*18*). Few published serosurveys in the United States include enough children to adequately describe SARS-CoV-2 infection in the pediatric population by subgroups. In addition, available state-level modeling data were not designed for subpopulation analysis within the pediatric population because of relatively low rates of pediatric hospitalization, limiting applicability to prevalence comparisons. 

We conducted a cross-sectional serosurvey to determine SARS-CoV-2 seroprevalence, seropositive case ascertainment, and age and racial/ethnic group differences during May–July 2021 in a convenience sample of children in Colorado, USA. The Colorado Department of Public Health and Environment (CDPHE) Communicable Disease Branch determined this activity to be consistent with enhanced disease surveillance activities, not human subjects research. Institutional review board approval was provided by the Colorado Multiple Institutional Review Board. Informed consent was waived.

## Methods

### Study Design and Conduct

Our cross-sectional serosurvey tested residual serum from a convenience sample of Colorado children 1–17 years of age from whom blood was collected during May 12–July 13, 2021, as part of care in nonsubspecialty outpatient clinics, urgent care centers, and emergency departments within the largest pediatric healthcare system in Colorado. We extracted age, sex, race, ethnicity, and specimen collection date from the electronic medical record. We assigned racial/ethnic group according to self-report as Hispanic/any race, non-Hispanic White, non-Hispanic Black, non-Hispanic other, and unknown. The non-Hispanic other group includes Asian, American Indian or Alaskan Native, Native Hawaiian or other Pacific Islander, >1 race, or other race. The unknown group includes persons for whom race and ethnicity data were not reported. For children included in the study, names and dates of birth were matched to the Colorado Immunization Information System to establish SARS-CoV-2 vaccination status and to the Colorado COVID-19 surveillance system to obtain SARS-CoV-2 molecular amplification test results reported to the CDPHE. Children for whom molecular amplification tests were positive were considered to have confirmed cases of COVID-19, per the Council of State and Territorial Epidemiologists surveillance case definition.

### Eligibility Criteria and Study Procedure

Residual serum specimens from children 1–17 years of age at the time of blood collection were eligible for inclusion. To improve generalizability, we excluded specimens from subspecialty outpatient clinics (e.g., oncology, cardiology). We selected eligible children sequentially. After selecting approximately half of the 1,000 planned specimens, we oversampled from the 1–4-year age group and non-Hispanic Black children to improve representativeness for subgroup analysis. To improve the sensitivity of positive serology results as a proxy for previous SARS-CoV-2 infection and to avoid misclassification error among previously vaccinated children not previously infected (Appendix Table 1), we excluded from final analysis children who had received >1 SARS-CoV-2 vaccine dose on any date before the serum specimen collection date. We also excluded non-Colorado residents from the final analysis.

### Laboratory Analysis

We tested residual serum specimens for SARS-CoV-2 nucleocapsid and spike IgG. We analyzed nucleocapsid antibodies by using the automated Abbott Alinity chemiluminescent microparticle immunoassay (Abbott, https://www.abbott.com). We determined the presence or absence of nucleocapsid antibodies by comparing the relative light units of the specimen (S) to a calibrator (C) and expressed results as an S/C index. We reported specimens with an S/C index <0.8 as negative, >1.4 as positive, and >0.8 to 1.3 as borderline for nucleocapsid antibodies. For purposes of this study, borderline results were considered negative.

We analyzed spike antibodies by using the qualitative Euroimmun ELISA Kit EI 2606–9601 (Euroimmun, https://www.euroimmun.com), which uses the S1 domain of the SARS-CoV-2 spike protein. We added specimens diluted 1:101 with dilution buffer, positive and negative controls, and kit-specific calibrator to SARS-CoV-2 S1 antigen precoated wells. We performed the ELISA according to the manufacturer’s specifications and read the final color development at optical density 450 nm (OD_450_). We interpreted results based on the ratio of the specimen OD_450_ to the calibrator OD_450_; specimens with a ratio of <0.8 were reported as negative, >1.1 as positive, and >0.8 to <1.1 as borderline for spike antibodies. For purposes of this study, we considered borderline results negative.

We established sensitivity and specificity of the Abbott and Euroimmun assays by using serum specimens with SARS-CoV-2 positivity confirmed by PCR and prepandemic specimens that had been collected before November 2019. According to our data, sensitivity of the Euroimmun assay was 92% and specificity 97%. We verified the manufacturer-stated sensitivity of the Abbott Alinity of 96.7% and specificity of 99% (*19*).

Reported viral tests used in the analysis included any molecular amplification test before the serum specimen collection date for each person. All viral test samples were collected, interpreted, and reported outside the context of this study.

### Statistical Analyses

We calculated descriptive characteristics, including counts and proportions with 95% CIs. We calculated SARS-CoV-2 seroprevalence by dividing the number of specimens positive for nucleocapsid IgG, spike IgG, or both by the total number of specimens tested among the sample of unvaccinated children (Appendix Table 2). We selected this method of calculating seroprevalence because it provides the most sensitive estimate of previous infection. To calculate sample prevalence of SARS-CoV-2 infection determined by confirmed cases reported to public health, we divided the number of persons with >1 positive molecular amplification test result preceding the serum specimen collection date by the total number of serum specimens tested. We included children with >1 previous positive molecular amplification test result only once. We calculated seropositive case ascertainment as the number of seropositive persons identified as confirmed cases reported to public health divided by the number of seropositive persons. We calculated multiplication factors, or the number of infections estimated per reported case, as the inverse of case ascertainment and prevalence ratios by racial/ethnic and age groups for serology results and case ascertainment. We analyzed differences in time between first positive viral test sample collection and serology sample collection stratified by serology test results by using Wilcoxon/Mann-Whitney testing. We conducted statistical analyses by using R version 4.1.1 (The R Foundation for Statistical Computing, https://www.r-project.org) and set statistical significance at p<0.05.

## Results

### Descriptive Characteristics

We collected 940 unique residual serum specimens for the study. We excluded 78 from children who had received >1 SARS-CoV-2 vaccine doses before specimen collection and 33 from children identified as non-Colorado residents. Among the 829 children included, 422 (50.9%) were female, and the median age was 9 (range 1–17) years (Table 1).

**Table 1:** Descriptive characteristics of children included in seroprevalence and case ascertainment analyses, Colorado, USA, May 12–July 13, 2021*

Characteristic	No. (%)	Median age (IQR)
Total population	829	9 y (3–14)
Female sex	422 (50.9)	NA
Male sex	405 (48.9)	NA
Racial/ethnic group		
Non-Hispanic White	290 (35.0)	8 (3.8–12)
Hispanic all races	287 (34.6)	10 (4–14)
Non-Hispanic Black	106 (12.8)	10.5 (3–14)
Non-Hispanic other†	99 (11.9)	8 (3–12)
Unknown‡	47 (5.7)	7 (2–13)
Age group, y		
1–4	254 (30.6)	NA
5–11	297 (35.8)	NA
12–17	278 (33.5)	NA

*IQR, interquartile range; NA, not applicable. †Includes persons self-reported as not Hispanic or Latino and Asian, American Indian or Alaskan Native, Native Hawaiian or other Pacific Islander, >1 race, or other race. ‡Includes persons for whom race and ethnicity data were not reported.

### SARS-CoV-2 Seroprevalence

Overall SARS-CoV-2 seroprevalence was 36.7% (95% CI 33.4%–40.1%) (Table 2). Seroprevalence across racial/ethnic groups was highest among Hispanic children at 49.8% (95% CI 43.9%–55.8%), followed by non-Hispanic Black at 37.7%, (95% CI 28.5%–47.7%) and non-Hispanic other race children at 35.4% (95% CI 26.0%–45.6%). Seroprevalence ratios showed higher seroprevalence for Hispanic (2.01 [95% CI 1.59–2.53]), non-Hispanic Black (1.52 [95% CI 1.11–2.08]), and non-Hispanic other race children (1.42 [95% CI 1.02–1.99]) than for non-Hispanic White children (referent group). Seroprevalence across age groups was 39.0% (95% CI 32.9%–45.3%) for children 1–4 years of age, 32.3% (95% CI 27.0%–38.0%) for children 5–11 years of age, and 39.2% (95% CI 33.4%–45.2%) for children 12–17 years of age. We found no statistically significant difference between age groups.

**Table 2:** SARS-CoV-2 serology results, seroprevalence, and seroprevalence ratios by racial/ethnic and age group, Colorado, USA, May 12-July 13, 2021*

Group	Serology results, no.		Seroprevalence, % (95% CI)	Seroprevalence ratio (95% CI)
	Seronegative	Seropositive†		
Total	525	304	36.7 (33.4–40.1)	NA
Race/ethnicity				
Non-Hispanic White	218	72	24.8 (20.0–30.2)	Referent
Hispanic all races	144	143	49.8 (43.9–55.8)	2.01 (1.59–2.53)‡
Non-Hispanic Black	66	40	37.7 (28.5–47.7)	1.52 (1.11–2.08)‡
Non-Hispanic other	64	35	35.4 (26.0–45.6)	1.42 (1.02–1.99)‡
Unknown	33	14	29.8 (17.3–44.9)	1.20 (0.74–1.94)
Age, y				
1–4	155	99	39.0 (32.9–45.3)	Referent
5–11	201	96	32.3 (27.0–38.0)	0.83 (0.66–1.04)
12–17	169	109	39.2 (33.4–45.2)	1.01 (0.81–1.24)

*NA, not applicable. †Seropositivity determined by presence of spike and/or nucleocapsid IgG. ‡Significant at p<0.05.

### Sample SARS-CoV-2 Prevalence Determined by Confirmed Case Counts 

Overall sample SARS-CoV-2 prevalence, determined by confirmed case counts reported to public health, was 6.5% (95% CI 4.9%–8.4%) (Appendix Table 3). Across racial/ethnic groups, SARS-CoV-2 prevalence by confirmed case counts was 7.6% (95% CI 4.8%–11.3%) for non-Hispanic White, 6.6% (95% CI 4.0%–10.2%) for Hispanic, and 3.8% (95% CI 1.0%–9.4%) for non-Hispanic Black children. Across age groups, SARS-CoV-2 prevalence by confirmed case counts was 5.1% (95% CI 2.8%–8.6%) for children 1–4 years of age, 7.4% (95% CI 4.7%–11.0%) for children 5–11 years of age, and 6.8% (95% CI 4.2%–10.5%) for children 12–17 years of age.

### Seropositive Case Ascertainment and Multiplication Factors

Seropositive case ascertainment in the overall study population was 16% with a multiplication factor of 6 (Table 3). Seropositive case ascertainment was highest among non-Hispanic White children (26.4% with multiplication factor of 4), followed by Hispanic (13.3%, multiplication factor of 8) and non-Hispanic Black children (7.5%, multiplication factor of 13). Seropositive case ascertainment prevalence ratios showed lower seropositive case ascertainment for Hispanic (0.50 [95% CI 0.28–0.89]) and non-Hispanic Black (0.28 [95% CI 0.09–0.90]) children than for non-Hispanic White children. Seropositive case ascertainment across age groups ranged from a high of 21.9% for the 5–11-year age group to a low of 13.1% for the 1–4-year age group. Case ascertainment prevalence ratios across age groups did not differ statistically.

**Table 3:** Previously reported positive SARS-CoV-2 viral diagnostic testing, seropositive case ascertainment, and multiplication factors by racial/ethnic and age group among seropositive children, Colorado, May 12–July 13, 2021*

Group	Previous positive result	Case ascertainment, % (95% CI)†	Case ascertainment prevalence ratio (95% CI)	Multiplication factor‡
Total	50	16.4 (12.5–21.1)	NA	6
Race/ethnicity				
Non-Hispanic White	19	26.4 (16.7–38.1)	Referent	4
Hispanic all races	19	13.3 (8.2–20.0)	0.50 (0.28–0.89§	8
Non-Hispanic Black	3	7.5 (1.6–20.4)	0.28 (0.09–0.90)§	13
Non-Hispanic other	6	17.1 (6.6–33.7)	0.65 (0.28–1.48)	6
Unknown	3	21.4 (4.7–50.8)	0.81 (0.28–2.38)	5
Age, y				
1–4	13	13.1 (7.2–21.4)	Referent	8
5–11	21	21.9 (14.1–31.5)	1.67 (0.89–3.13)	5
12–17	16	14.7 (8.6–22.7)	1.12 (0.57–2.21)	7

*NA, not applicable. †Case ascertainment calculated as the number of previous positive tests among seropositive children divided by the total number of seropositive children. ‡Multiplication factor calculated as the number of seropositive cases divided by the number of previous positive tests, also known as the inverse of case ascertainment. §Statistically significant at p<0.05.

### Spike Versus Nucleocapsid IgG Results

**Figure 1:**
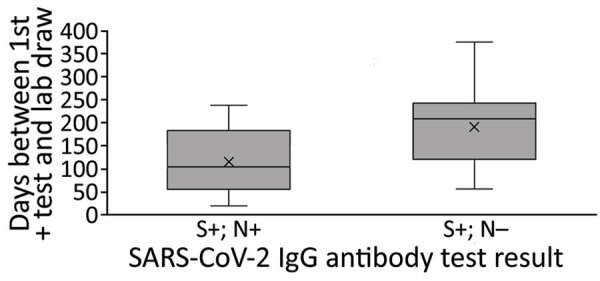
Days between first positive diagnostic test result and sample collection for serology testing for SARS-CoV-2 IgG, Colorado, USA, May 12–July 13, 2021. N, nucleocapsid; S, spike.

Of the 54 children with a previous reported positive SARS-CoV-2 viral test result, 36 (66.7%) were seropositive for both spike and nucleocapsid IgG, 13 (24.1%) for spike IgG only, 4 (7.4%) for spike and nucleocapsid IgG, and 1 (1.9%) for nucleocapsid IgG only. The average interval between the time of the first positive SARS-CoV-2 viral test specimen collection and serum specimen collection was 116 days for those seropositive for spike and nucleocapsid IgG and 191 days for those seropositive for spike IgG only (p = 0.005) (Figure 1).

## Discussion

In this convenience sample of residual serum collected from Colorado children 1–17 years of age during spring/summer 2021, seroprevalence of SARS-CoV-2 IgG was 36.7%. Our results show higher seroprevalence than that found from rounds 20 and 21 of the nationwide antibody seroprevalence survey (https://covid.cdc.gov/covid-data-tracker/#national-lab), the only contemporaneous rounds with an estimate for seroprevalence among children, which estimated seroprevalence of 13.4% (95% CI 6.2%–23.8%) for round 20 and 17.5% (95% CI 12.4%–22.8%) for round 21 (*20*). A few factors may explain those differences: the nationwide surveys were conducted just before and during the first few weeks of our study, the nationwide survey used a different nucleocapsid assay and did not test for spike antibodies, and the relatively small pediatric populations in these convenience samples may demographically differ from those in our sample. In addition, although adjusted rates, such as those calculated in the nationwide survey, are comparable to other similarly adjusted rates across different populations, they may not be comparable to unadjusted rates such as those in our study. However, results for round 25, conducted a few months after the conclusion of our sample selection, was much more consistent with our results: seroprevalence of 40.1% (95% CI 33.7%–46.6%).

Among our study cohort, SARS-CoV-2 prevalence determined by positive molecular amplification testing was only 6.5% and seropositive case ascertainment was 16%. This finding indicates a potential underestimation of cases among this pediatric population by 84% when relying solely on confirmed case counts, either because of underascertainment or underreporting. If the unadjusted study results were generalized to the larger Colorado pediatric population, confirmed case counts statewide could require a multiplication factor of 6 to more accurately reflect the prevalence of SARS-CoV-2 infection among children. This result is consistent with findings from other states that showed multiplication factors from 4.7 to 8.9 during a similar time frame (*21*). Our results may represent opportunities to improve individual-level awareness of SARS-CoV-2 infection and increase mitigation measures, including isolation and quarantine. On a population level, our results can be used to corroborate modeled SARS-CoV-2 prevalence estimates based on hospitalization rates.

In addition, we found statistically significant differences in seroprevalence and seropositive case ascertainment across racial/ethnic groups. Compared with non-Hispanic White children, seroprevalence was significantly higher among Hispanic and non-Hispanic Black children, indicating higher rates of previous SARS-CoV-2 infection, consistent with results of several published studies on molecular test positivity and seroprevalence studies in adults (*11*,*22*–*27*). Our study also found that seropositive case ascertainment was lower among Hispanic and non-Hispanic Black children than among non-Hispanic White children; cases were detected in Hispanic children half as often as in non-Hispanic White children, and cases in non-Hispanic Black children were detected less than one third as often as in non-Hispanic White children. This disparity is supported by studies showing reduced rates of testing in many racial/ethnic minority groups (*22*,*27*). Our results show that reliance on reported case counts would lead to the erroneous assumption that rates of SARS-CoV-2 infection in the study period were highest among non-Hispanic White children. Those results further demonstrate the value of complementary surveillance mechanisms such as serologic and wastewater testing that can avoid testing and reporting biases.

Seroprevalence measured in our study reflects infections among children occurring during the 2020–21 school year and early summer before the peaks of the Delta and Omicron variants in Colorado. During that period, several school-based SARS-CoV-2 mitigation measures were mandatory in Colorado (e.g., masking and classroom cohorting), and in-person learning was interspersed with remote learning for intermittent school closures or hybrid learning models. Despite those measures and questions of whether children were at lower risk for infection with and transmission of SARS-CoV-2, compared with reported viral test results, our study shows that SARS-CoV-2 prevalence among children was higher. Those findings may have broad implications for estimating the severity of SARS-CoV-2 among children, levels of potential immunity, and the effectiveness of various mitigation measures in the pediatric population, and they may help guide future public health recommendations.

**Figure 2:**
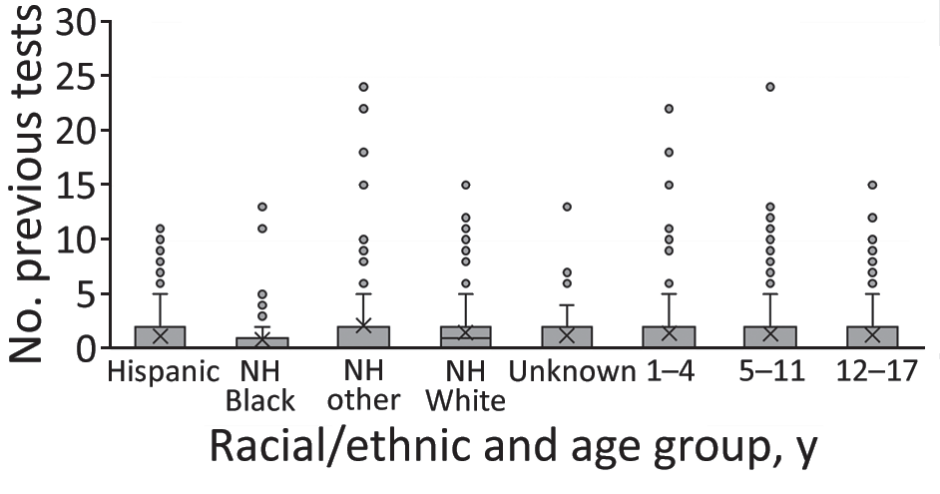
Boxplots showing number of previous tests, by racial/ethnic and age groups, among 829 children included in seroprevalence and case ascertainment analyses, Colorado, USA, May 12–July 13, 2021. NH, non-Hispanic.

Our study did not evaluate causal mechanisms for the observed racial/ethnic disparities in previous SARS-CoV-2 infection or seropositive case ascertainment. However, those findings could be explained by the existing literature, which suggests differences in risk for exposure to SARS-CoV-2 because of occupation or living setting, test-seeking behavior, underlying health and healthcare disparities, or differential barriers to viral testing (*22*,*28*). Despite extensive efforts at the federal, state, and local levels to improve access to free SARS-CoV-2 testing throughout Colorado during the study period, some barriers to access (e.g., time and transportation) may persist in some communities. In addition, differences in individual factors (e.g., perceived vulnerability and variability in access to childcare) could guide testing-seeking behaviors. The discrepancy between the number of seropositive children with any prior test (162, 53.3%; Appendix Table 3) and the number with previous positive test results (50, 16.4%) suggests that timing of viral testing or changes in access over time may also complicate case identification efforts. That 53% of seropositive persons had been previously tested but only 16% ever received a positive test result suggests that access to testing is necessary but not sufficient for identifying cases. The overall number of previous tests was skewed toward zero across all racial/ethnic and age groups; however, each group had outliers with high numbers of prior testing (Figure 2). The only group with a median number of tests >0 was non-Hispanic White children. To improve case finding within this population, greater knowledge of drivers for and access to viral testing is needed. Among children, for whom cases are often asymptomatic or mildly symptomatic, pursuing regular screening and postexposure testing may be needed to improve case ascertainment or better incorporate pediatric specimens in serosurveys to provide more information about disease prevalence. These considerations may also apply to other populations with largely asymptomatic illness, possible future SARS-CoV-2 variants that may cause fewer symptoms, or emergence of novel respiratory viruses with similar symptoms in children.

Results from this study are also consistent with literature suggesting that the SARS-CoV-2 spike IgG response may be more durable than the nucleocapsid IgG response because children who were spike IgG positive but nucleocapsid IgG negative were further from their positive test result at the time of serum sampling (*29*–*34*). As with any laboratory test, knowledge of the test characteristics, including seroreversion over time in the case of serology tests, is relevant for study design and interpreting results. The differential kinetics of IgG response after SARS-CoV-2 infection require further study, should inform serosurveillance efforts, and may complicate the ability to estimate SARS-CoV-2 prevalence among vaccinated populations.

Among the limitations of this study, the use of reported COVID-19 PCR and other molecular viral testing may miss some positive results from viral tests, such as antigen tests and testing performed out of state. However, during the study period, antigen tests were not widely available in Colorado, and molecular tests were the recommended test for schools. Free testing services provided by the state throughout Colorado used PCR testing, as did other large testing providers serving schools during the study period. Similarly, use of the Colorado Immunization Information System to determine vaccination status might lead to misclassification of some children as unvaccinated, although the time between specimen collection and data analysis should greatly limit the potential for missing vaccine data.

Second, unadjusted results from the study convenience sample may not be generalizable to other pediatric populations. Exclusion of previously vaccinated children could introduce some bias because only certain age groups were eligible for vaccination and vaccinated children may differ in relevant ways from their unvaccinated peers. Specifically, half of excluded vaccinees were non-Hispanic White children, and all were 12–17 years of age. However, we believe that exclusion of this group (Appendix Table 1) is likely to bias the sample toward the null and may lead to underestimation of the disparities noted. Although residence was not included in the study, this convenience sample may overrepresent children from the Denver-metro area, who account for a larger percentage of the patient population in this healthcare system, reducing generalizability. Because specimens were selected from children within a large pediatric healthcare system, this study cohort may represent children with greater access to care and families who are more likely to seek care, which could be associated with fewer barriers to testing. This population may also have a greater number of underlying health conditions, although our study sought to limit some of this bias by excluding children from subspecialty care clinics, which could lead to higher case ascertainment in this than in other populations. Similarly, SARS-CoV-2 positivity may be higher in the study cohort because symptomatic children seeking care and for whom providers ordered serum-based laboratory tests may be overrepresented, which could lead to higher seroprevalence.

Third, because of low numbers of members of certain racial/ethnic groups, including children in the non-Hispanic Asian, American Indian or Alaskan Native, Native Hawaiian or other Pacific Islander, >1 race, or other race categories, we were unable to analyze completely disaggregated racial/ethnic groups. This limitation is notable because there are known health disparities in some of the aggregated racial/ethnic groups, and a sample powered for analysis of these groups may identify similar disparities in SARS-CoV-2 serologic results and case ascertainment.

Last, seroconversion is an imperfect proxy for measuring disease prevalence because not all persons seroconvert after SARS-CoV-2 infection, and some literature suggests that rates of seroconversion may be lower or that antibody waning after infection may be more rapid for children or those with asymptomatic or mild illness (*34*–*38*). However, because some data on this issue are conflicting, the potential effect on our results is unclear (*39*). Similarly, the exact test characteristics over time for the assays used are not well known.

Strengths of the study include our ability to individually match serologic results to associated vaccination and reported test results, which enabled us to make direct comparisons and avoids ecological fallacy, which can arise when inferring information about individuals based on results from population-based data. Also, oversampling of children 1–4 years of age and Black children improved the representativeness of our sample across subgroups, which enabled a more nuanced analysis of racial/ethnic differences in serologic results and case ascertainment. 

In conclusion, before the Delta variant peak, we found evidence of previous SARS-CoV-2 infection in more than one third of Colorado children 1–17 years of age based on seroprevalence, despite far fewer confirmed cases in this study population. Repeating this analysis after the Delta and Omicron variant peaks would be prudent. Racial/ethnic disparities identified in this pediatric population are consistent with trends identified among adults; higher seroprevalence, lower rates of reported viral testing, and lower seropositive case ascertainment were found among Hispanic and Non-Hispanic Black children than among non-Hispanic White children. Continued efforts to address racial/ethnic differences in illness burden and potential barriers to viral testing in pediatric populations are warranted.

Appendix Supplemental results from study comparing SARS-CoV-2 seroprevalence with confirmed COVID-19 cases among children, Colorado, USA, May–July 2021.
