# Supplementary material for: SARS-CoV-2 Seroprevalence Compared with Confirmed COVID-19 Cases among Children, Colorado, USA, May–July 2021
Source: Emerg Infect Dis. 2023 May;29(5):929–36. doi: 10.3201/eid2905.221541 (PMC10124638; doi:10.3201/eid2905.221541)
Supplement: Appendix — Supplemental results from study comparing SARS-CoV-2 seroprevalence with confirmed COVID-19 cases among children, Colorado, USA, May–July 2021. [file 22-1541-Techapp-s1.pdf]

Article DOI: https://doi.org/10.3201/eid2905.221541

EID cannot ensure accessibility for supplementary materials supplied by authors. Readers who have difficulty accessing supplementary content should contact the authors for assistance.

SARS-CoV-2 Seroprevalence Compared with Confirmed COVID-19 Cases among Children, Colorado, USA, May–July 2021

Appendix

Appendix Table 1. Descriptive characteristics of 78 previously vaccinated children excluded from subsequent analysis, Colorado, May 12-July 13, 2021

Characteristic	No. (% of excluded population)
Previously vaccinated (% of total population)	78 (8.3)
1 Prior vaccination	26 (33.3)
2 Prior vaccinations	52 (66.7)
Spike IgG Ab results	
Positive	67 (85.9)
Negative	11 (14.1)
Nucleocapsid IgG Ab results	
Positive	11 (14.1)
Negative	66 (84.6)
Not performed	1 (1.3)
Racial-ethnic group	
Non-Hispanic White	39 (50.0)
Hispanic all races	17 (21.8)
Non-Hispanic Black	8 (10.3)
N Non-Hispanic other	9 (11.5)
Unknown	5 (6.4)
Age group, y	
1–4	0 (0)
5–11	0 (0)
12–17*	78 (100)

*Only 12–17 y olds were eligible for vaccination during this period.

Appendix Table 2. Combined serology results for 829 children included in seroprevalence and case ascertainment analyses, Colorado, May 12-July 13, 2021

	Nucleocapsid IgG Ab result, no. (%)			
Spike IgG Ab Result	Positive	Negative	Not performed	Total
Positive	145 (17.5)	153 (18.5)	1 (0.1)	299 (36.1)
Negative	5 (0.6)	521 (62.8)	4 (0.5)	530 (63.9)
Total	150 (18.1)	674 (81.3)	5 (0.6)	829 (100)

Appendix Table 3. Previously reported SARS-CoV-2 viral diagnostic testing by race//ethnicity, age group, and IgG results among 829 children included in seroprevalence and case ascertainment analyses, Colorado, USA, May 12-July 13, 2021

	Any previous test*, no. (%)		Previous positive test, no. (%)		Population total, no.	
Group	Among seropositive children	Among all children	Among seropositive children	Among all children	Seropositive children	All children
Total	162 (53.3)	392 (47.3)	50 (16.4)	54 (6.5)	304	829
Racial/ethnic group						
Non-Hispanic White	46 (63.9)	147 (50.7)	19 (26.4)	22 (7.6)	72	290
Hispanic all races	70 (49.0)	138 (48.1)	19 (13.3)	19 (6.6)	143	287
Non-Hispanic Black	18 (45.0)	39 (36.8)	3 (7.5)	4 (3.8)	40	106
Non-Hispanic other	20 (57.1)	49 (49.5)	6 (17.1)	6 (6.1)	35	99
Unknown	8 (57.1)	19 (40.4)	3 (21.4)	3 (6.4)	14	47
Age group, y						
1–4	50 (50.5)	119 (46.9)	13 (13.1)	13 (5.1)	99	254
5–11	61 (63.5)	143 (48.1)	21 (21.9)	22 (7.4)	96	297
12–17	51 (46.8)	130 (46.8)	16 (14.7)	19 (6.8)	109	278
IgG Ab result						
S+;N+	83 (57.2)	-	36 (24.8)	-	145	-
S+;N–	75 (49.0)	-	13 (8.5)	-	153	-
S–;N+	3 (60.0)	-	1 (20.0)	-	5	-

*Any previous test includes positive and negative reported viral test results.
